# Current medical education improves OSA-related knowledge but not confidence in residents: An underappreciated public health risk

**DOI:** 10.3389/fpsyt.2022.973884

**Published:** 2022-11-11

**Authors:** Linfan Su, Ruxuan Chen, Jinmei Luo, Yi Xiao

**Affiliations:** Department of Pulmonary and Critical Care Medicine, Peking Union Medical College Hospital, Chinese Academy of Medical Sciences, Peking Union Medical College, Beijing, China

**Keywords:** medical education, survey and questionnaires, residency training, obstructive sleep apnea, training effect

## Abstract

**Background:**

Obstructive sleep apnea (OSA) is the most common sleep-related breathing disorder and induces a growing health care burden. However, a large proportion of patients with OSA do not receive appropriate treatment and are underdiagnosed or misdiagnosed in primary care. A contributing factor to the phenomenon is the lack of education, which reflects the current inadequacies in medical education. Therefore, assessing the level of knowledge and attitudes toward OSA and associated factors among resident physicians is highly warranted.

**Methods:**

A validated questionnaire, the OSA Knowledge and Attitudes (OSAKA) questionnaire was distributed to residents who had already completed undergraduate education and were attending an internal medicine residency training program. The questionnaire consists of 2 parts: including an assessment of (1) OSA-related knowledge involving epidemiology, pathophysiology, clinical manifestations, diagnosis, and treatment; (2) the importance of OSA and confidence in diagnosing and treating OSA patients. Other information including demographics, training experience, and questions exploring the future form of the sleep breathing disorder course was collected together.

**Results:**

Of the 160 residents who participated in the survey, 153 (95.6%) completed the survey and the mean total knowledge score was 12.6/18 (70% correct). Although all respondents believed that OSA was an important clinical disorder, only a minority of the residents felt confident in identifying patients at risk for OSA (38%), managing OSA patients (27.5%), or continuous positive airway pressure therapy (CPAP) (26.2%). We found that OSA training experience significantly increased knowledge scores (*p* = 0.002) but not confidence scores (*p* = 0.248). As for the specific form of medical education, “Small classes during residency training” was the most popular form of sleep-breathing disorder educational training in the future of the resident training program.

**Conclusion:**

Despite adequate knowledge of OSA, there was still a generalized lack of confidence in the management of OSA patients among residents. Current medical education can not build enough confidence for physicians, which may in turn affect patients' trust and reduce long-term compliance. Untreated OSA places a significant health threat and economic burden on not only the patients but also their families and society, causing an underappreciated public health risk. In the future, merely increasing OSA courses is not sufficient, a more specific focus on the course format and training effect is required.

## Introduction

Obstructive sleep apnea (OSA) is a highly prevalent sleep-related breathing disorder characterized by repetitive obstruction of the pharyngeal airway during sleep, causing nocturnal hypoxemia and fragmented sleep (1). It has been estimated that 936 million adults worldwide aged 30–69 years suffer from OSA (2). However, most OSA patients remain underdiagnosed, untreated, or undertreated in primary care mainly because of the cumbersome and time-consuming diagnostic and treatment process (3). With the ongoing obesity epidemic, the estimated prevalence of OSA represents substantial increases over decades (4). Untreated OSA can negatively affect multiple systems in the long term, leading to hypertension, coronary heart disease, stroke, diabetes, and neurocognitive abnormalities (5–7). Therefore, OSA is a very significant public health issue and has been included in chronic disease management programs, which requires clinical attention, especially from general practitioners and other primary care physicians (8, 9).

Despite the increased risk for sleep disorders among minority or medically indigent individuals, in some community settings, the diagnosis rates of OSA were <1% (10). A contributing factor to the phenomenon that sleep disorders are commonly underdiagnosed among health care providers is the lack of education (11, 12). It is believed that improving physicians' knowledge about OSA is critical for improving OSA-related screening and treatment practices (13, 14). More time needs to be devoted to education on sleep disorders in medical school curricula. However, instruction on sleep and sleep disorders during medical education and training remains limited over the years (15, 16). A survey across 12 countries found that the average time spent on sleep education is just under 2.5 h, with nearly one-third of medical school do not provide sleep education (11). Although corresponding guidelines for the management of OSA among physicians have been issued around the world, their effect on clinical work is insufficient as more physicians need related practice (17, 18). At present, unified curriculums or requirements for sleep education medical residency training programs do not exist. A recent survey revealed that absent sleep medicine training in most residency training is limited in the US (12). To date, no such study has been conducted or reported in China.

As the first step of clinical training for medical students, residency training is a unique opportunity for resident physicians to improve basic clinical skills and has been promoted nationally over the last decade (19, 20). Serve as a unique window, residency training programs provide opportunities for the residents to formally expose to sleep medicine. Since exposure is associated with subspecialty choice, increasing resident exposure may help to improve the current workforce shortage in sleep medicine (21), which is associated with many health risks. The medical fields of internal medicine, family medicine otolaryngology, psychiatry, and neurology are deemed to be the most important for knowledge regarding sleep disorders (12). Among them, internal medicine physicians frequently come into contact with OSA patients and their chronic complications (22). Thus, it makes more sense to investigate residents in the internal medicine residency training program, who are more likely to be responsible for the OSA diagnosis and treatment upon completion of their training.

Peking Union Medical College Hospital (PUMCH, an experienced residential training base) is the top hospital in China and has the oldest history and abundant experience in residency training programs throughout the country (23). Investigating the knowledge and attitudes of residents in PUMCH could help improve the national resident training program. Therefore, the purposes of this study were to understand the current status of OSA education among residents and their ability to identify and manage OSA patients, which may be helpful for future education improvement.

## Materials and methods

### Study design and ethics approval

This was a cross-sectional survey conducted among resident physicians during residency training programs in PUMCH between December 2019 and June 2020. The study was approved by the Medical Ethics Committee of Peking Union Medical College Hospital (Approval No. S-K 954). Informed consent was obtained from all individual participants included in the study.

### Study participants and data collection

We recruited residents who were attending an internal medicine residency training program in PUMCH at the time of the survey. By querying the registration information of the hospital education office, we obtained the contact details of all resident physicians. Eligible physicians (*n* = 160) were contacted by phone to obtain permission to send them the study questionnaire. An informed consent form and questionnaire links were sent to participants' phones *via* WeChat. Data was collected *via* Wenjuanxing software. Residents were excluded from the study if they declined to participate (*n* = 7). Finally, 153 questionnaires were collected. Participation was voluntary.

A self-administered questionnaire which included the Chinese version of the Obstructive Sleep Apnea Knowledge and Attitude (OSAKA) questionnaire was used to collect data. OSAKA has been translated from English into Chinese version by two researchers with repeated revisions independently to ensure translation accuracy. All doctors attending the internal medicine residency training programs were invited to participate in the study, including internal medicine residents, general medicine residents, and others. According to their residency training years, the residency was classified into postgraduate years (PGY) 1, 2, and 3. And age was categorized into 3 groups (<25 years, 25–30 years, and >30 years).

### Survey structure

The validated OSAKA questionnaire was used to evaluate the knowledge, attitudes, and confidence of PUMCH residents about OSA (13). The questionnaire was divided into three parts.

1. The first part evaluated basic OSA knowledge involving epidemiology (Q3, Q13, Q15), pathophysiology (Q9–10, Q12), symptoms (Q1, Q4–5, Q18), and diagnosis (Q6, Q11, Q14, Q17), and treatments (Q7–8, Q2, Q16). There were 18 questions with “True,” “False,” and “Unsure/Do Not Know” options, and the generated total scores ranged from 0 to 18. Correct responses scored 1 point, while incorrect and “Unsure/Do Not Know” answers received 0 points.

2. The second part assessed the attitudes of two sections: the importance of OSA and confidence in diagnosing and treating OSA patients. The questionnaire used the five-point Likert scale. Sections on importance ranged from 1 (not important) to 5 (extremely important), and those on confidence ranged from 1 (strongly disagree) to 5 (strongly agree). Additionally, demographic data, including participants' age, gender, and level of training, were also collected.

3. The last part included two additional questions investigating the willingness and the future form of the sleep breathing disorder course. Multiple-choice questions were used to show residents' preferences.

### Statistical analyses

Statistical calculations were performed using SPSS software (version 25). GraphPad Prism9 were used for chart production. Descriptive analyses were calculated for demographic characteristics. We summarized all categorical variables as proportions (%) and numbers (n) and described continuous variables as means and standard deviation (M ± SD). The association between demographic characteristics and OSA knowledge and attitude scores was examined using two-sample t-test and oneway-ANOVA for continuous variables. ANOVA was followed by *post hoc* LSD (Least Significant Difference) test to determine whether there is a significant difference between groups, as appropriate. Pearson's correlation was used to determine the relationship between the knowledge scores and the attitude scores. To identify independent determinants of the total knowledge scores and attitude scores, we used multivariable linear regression models. A *p*-value of < 0.05 was considered statistically significant.

## Results

### Participants characteristics

A total of 153 questionnaires were received from 160 residents with a response rate of 95.63%. The basic information is shown in Table 1. Approximately two-thirds of the respondents (64.1%) were aged 25–30 years, and the majority (71.9%) were females. Most of the respondents had the highest education level of a doctoral degree (54.9%), followed by a master's (32.7%) and a bachelor's degree (12.4%). Residents who specialized in internal medicine represented about 82.4%, constituting the majority. More than half of the residents (57.5%) had not participated in OSA training before. There were 38.6% of the residents denied the experience of treating OSA patients.

**Table 1:** Demographic characteristics of the participants (*N* = 153).

**Variables**	**Characteristic**	***N***	**(%)**
**Age (years)**	<25	46	(30.1)
	25-30	98	(64.1)
	>30	9	(5.9)
**Gender**	Male	43	(28.1)
	Female	110	(71.9)
**Highest level of education**	Bachelor	19	(12.4)
	Master	50	(32.7)
	Ph.D. or MD	84	(54.9)
**Specialty**	Internal medicine	126	(82.4)
	General medicine	7	(4.6)
	Other	20	(13.1)
**Level of training**	PGY 1	53	(34.6)
	PGY 2	49	(32.0)
	PGY 3	51	(33.3)
**Had OSA training**	Yes	65	(42.5)
	No	88	(57.5)
**Treated OSA patients**	Yes	53	(34.6)
	No	59	(38.6)
	Unsure	41	(26.8)

Ph.D., Philosophy Doctor; MD, Medical Doctor; PGY, Post Grad Year.

### Knowledge of OSA

**Figure 1:**
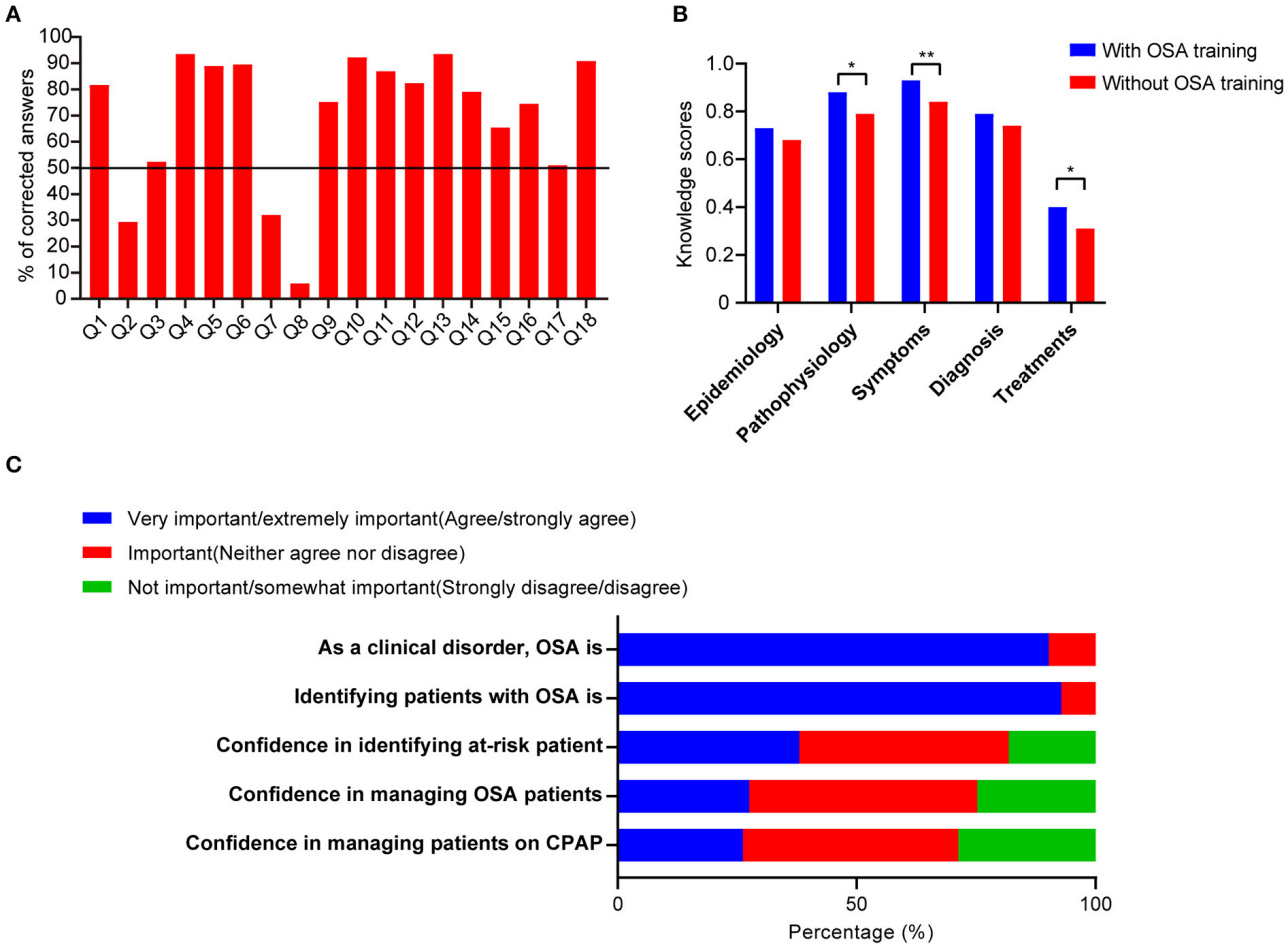
Results of knowledge and attitude scores. **(A)** Percentage of corrected answers among the knowledge items. **(B)** The bar graph demonstrated a comparison of knowledge scores related to different characteristics among residents who had OSA training education or not. **(C)** Comparison of the composition of the responses in the attitude scores. *p < 0.05, **p < 0.01.

The average knowledge score for all respondents was 12.6 ±2.7 (70% correct, Figure 1A and Table 2). We calculated separately the correct percentage of respondents for each part of OSA knowledge. In general, the correct rate of treatment part (35.5%) is lower than 50%, while pathophysiology (83.2%), symptoms (88.7%), diagnosis (76.6%), and epidemiology (70.4%) are higher than 50%. Compared with residents without OSA training, residents with previous OSA training have higher knowledge scores related to the pathophysiology (*p* = 0.015), symptoms (*p* = 0.001), and treatments (*p* = 0.022) in Figure 1B and Supplementary Table S1.

**Table 2:** Specific items of knowledge questions and the proportion of correct answers (*N* = 153).

**Knowledge questions**	**Correct answer**	**Number of correct responses**	
		**N**	**(%)**
Q1. Women with OSA may present with fatigue alone	True	125	(81.7)
Q2. Uvulopalatopharyngoplasty is curative for a majority of people with OSA	False	45	(29.4)
Q3. The estimated prevalence of OSA among adults is between 2 and 10%	True	80	(52.3)
Q4. The majority of patients with OSA snore	True	143	(93.5)
Q5. OSA is associated with hypertension	True	136	(88.9)
Q6. An overnight sleep study is the gold standard for diagnosing OSA	True	137	(89.5)
Q7. CPAP (continuous positive airway pressure) therapy may cause nasal congestion	True	49	(32.0)
Q8. Laser-assisted uvuloplasty is an appropriate treatment for severe OSA	False	9	(5.9)
Q9. The loss of upper airway muscle tone during sleep contributes to OSA	True	115	(75.2)
Q10. The most common cause of OSA in children is the presence of large tonsils and adenoids	True	141	(92.2)
Q11. A craniofacial and oropharyngeal examination is useful in the assessment of patients with suspected OSA	True	133	(86.9)
Q12. Alcohol at bedtime improves OSA	False	126	(82.4)
Q13. Untreated OSA is associated with a higher incidence of automobile crashes	True	143	(93.5)
Q14. In men, a collar size 17 inches or greater is associated with OS.	True	121	(79.1)
Q15. OSA is more common in women than in men	False	100	(65.4)
Q16. CPAP is the first line of therapy for severe OSA	True	114	(74.5)
Q17. Less than 5 apneas or hypopneas per hour is normal in adults	True	78	(51.0)
Q18. Cardiac arrhythmias may be associated with untreated OSA	True	139	(90.8)
**Total OSA Knowledge score [mean (SD)]**		12.6	(2.7)

OSA, obstructive sleep apnea, CPAP, continuous positive airway pressure.

### Attitude toward OSA

Overall, the average OSAKA attitude score for all respondents was 3.6 ± 0.2 and detailed results are shown in Supplementary Table S2. Residents with different seniorities all considered OSA as an important clinical disorder and regarded it as important to identify suspected OSA patients. Regarding the confidence in identifying patients at risk for OSA, ability to manage OSA patients, and manage patients on continuous positive airway pressure (CPAP) therapy, favorable confidence scores (agree/strongly agree) were reported only in 38.0%, 27.5%, and 26.2% of all respondents, respectively (Figure 1C).

### Analysis of differences between groups

Younger residents (<25 years old) had a lower average knowledge score (11.6 ±3.3) when compared with the older residents (25–30 years old, 13.0 ± 2.3; > 30 years old, 13.1 ± 3.0). There were no statistically significant differences between specialty or education level (Table 3). As expected, junior residents' average knowledge scores (PGY1, 11.7 ± 3.2) were significantly lower compared to senior residents' (PGY2, 13.1 ± 1.8; PGY3, 13.1 ± 2.7). Residents who had attended OSA training or treated OSA patients before had higher knowledge scores (*p* < 0.05). Aside from age, there were no significant differences observed in gender, the highest level of education, level of training, and OSA training experience in attitude scores.

**Table 3:** Association between demographic characteristics and means of OSA knowledge and attitude score.

**Variables**	**Knowledge score**		**Attitude score**	
	**Mean (SD)**	***p-*value**	**Mean (SD)**	***p-*value**
**Age (years)**		**0.009**		**0.001**
<25	11.6(3.3)	0.003^*^	3.3(0.6)	<0.001^*^
25–30	13.0(2.3)	0.940^†^	3.7(0.6)	0.530^†^
>30	13.1(3.0)	0.120^‡^	3.8(0.7)	**0.020** ^‡^
**Gender**		**0.017**		0.430
Male	11.8(3.4)		3.5(0.8)	
Female	12.9(2.4)		3.6(0.6)	
**Highest level of education**		0.196		0.282
Bachelor	13.3(2.2)		3.5(0.7)	
Master	12.9(2.5)		3.7(0.6)	
Ph.D. or MD	12.3(2.9)		3.6(0.7)	
**Specialty**		0.050		0.511
Internal medicine	12.8(2.7)		3.6(0.6)	
General medicine	10.3(3.1)		3.3(0.6)	
Other	12.3(2.9)		3.6(0.8)	
**Level of training**		**0.013**		0.065
Post grad year 1	11.7(3.2)	**0.012^§^**	3.5(0.7)	
Post grad year 2	13.1(1.8)	0.945 ^||^	3.7(0.6)	
Post grad year 3	13.1(2.7)	**0.009** ^¶^	3.7(0.6)	
**Had OSA training**		**0.002**		0.591
Yes	13.4(2.4)		3.7(0.6)	
No	12.0(2.8)		3.6(0.7)	
**Treated OSA patients**		**0.003**		0.445
Yes	13.3(2.5)	0.002 ^**^	3.6(0.5)	
No	11.7(3.0)	**0.011 ^††^**	3.5(0.8)	
Unsure	13.1(2.2)	0.704 ^‡‡^	3.7(0.6)	

* p-value: <25 vs. 25–30;

† p-value: 25–30 vs.>30;

‡ p value: <25 vs. >30;

§ p value:PGY1 vs. PGY2;

|| p-value:PGY2 vs. PGY3;

¶ p value:PGY1 vs. PGY3;

** p-value: Yes vs. No;

†† p value: no vs. unsure;

‡‡ p value: yes vs. unsure. Significant differences are in bold.

### Factors influencing knowledge and attitude scores

**Figure 2:**
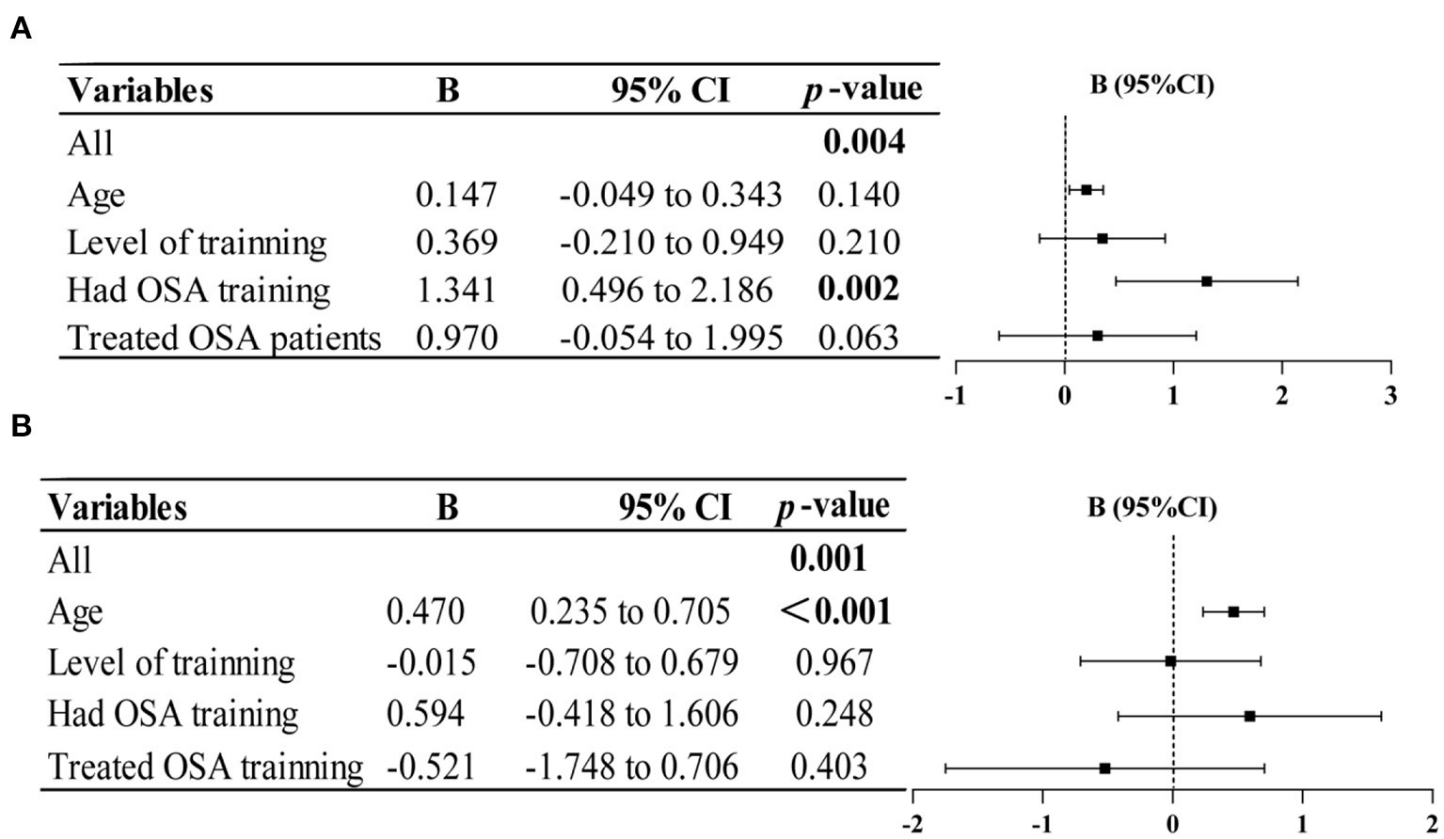
Multiple linear regression analysis revealed factors influencing knowledge **(A)** and attitude scores **(B)**. Significant differences are in bold.

We found several variables had a statistically significant correlation with knowledge scores and attitude scores (Table 4). Further validation was performed using multivariate linear regression analysis to explore the independent determinants. The result indicates that knowledge scores are only significantly positively correlated with OSA training experience (*p* = 0.002). While attitude scores are only significantly positively correlated with age (*p* < 0.001) (Figure 2).

**Table 4:** Correlation analysis of the knowledge score and the attitude score.

**Variables**	**Knowledge score**		**Attitude score**	
	**r**	***p*-value**	**r**	***p*-value**
Age	0.217	**0.007**	0.337	**< 0.001**
Gender	0.113	0.117	0.064	0.430
Highest level of education	−0.140	0.085	−0.079	0.334
Specialty	−0.122	0.133	−0.025	0.755
Level of training	0.210	**0.009**	0.171	**0.035**
Had OSA training	0.250	**0.002**	0.044	0.591
Treated OSA patients	0.254	**0.002**	0.059	0.472

Significant differences are in bold.

### Training for OSA

**Figure 3:**
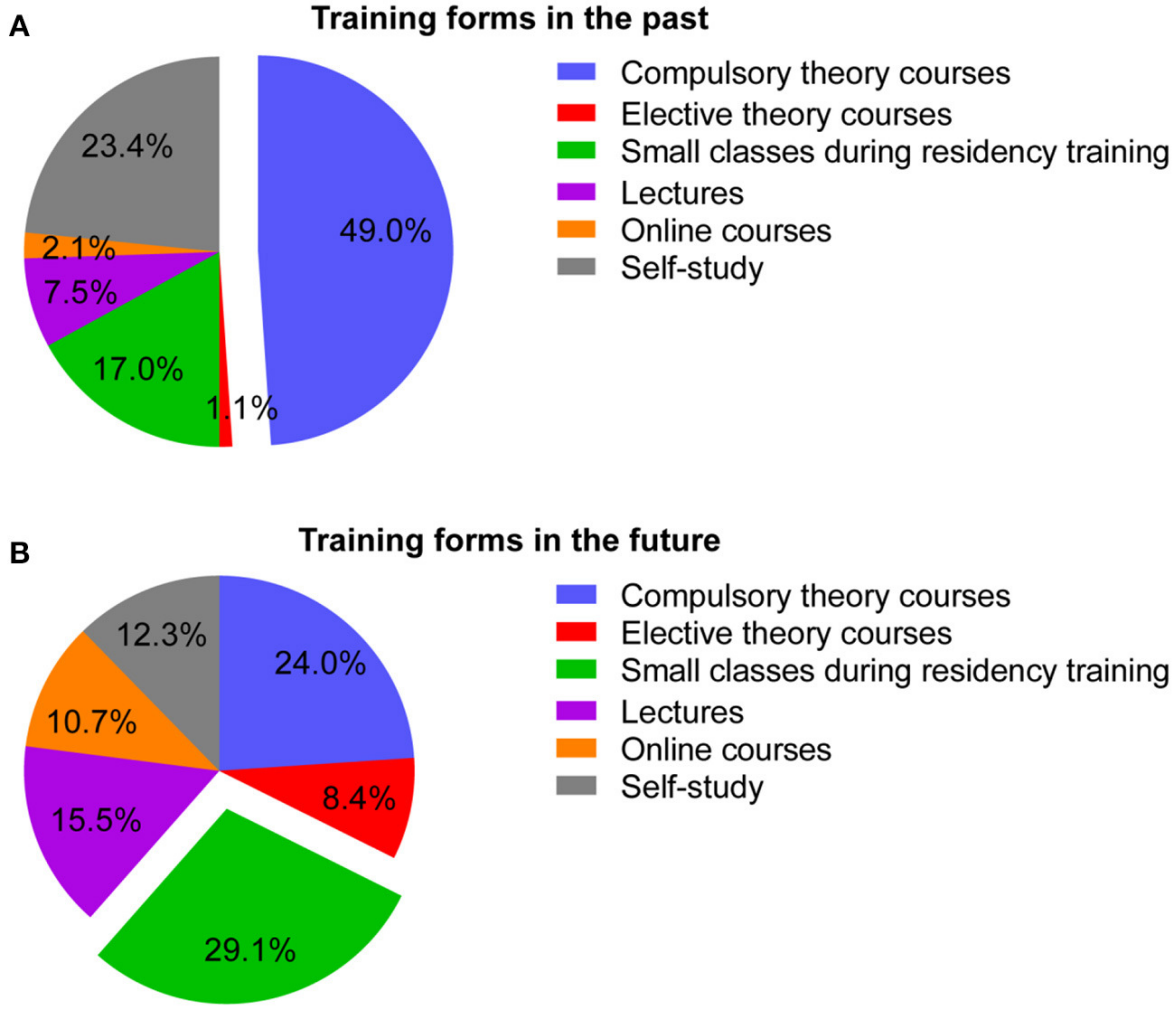
Composition of the form of the sleep breathing disorder course received in the past **(A)** and chosen in the future **(B)**.

To enhance physicians' recognition of OSA's role in clinical practice, we performed an exploratory survey to investigate the form of the sleep breathing disorder course received in the past and future in detail (Figure 3). The main source of previous OSA training is compulsory theory courses (49.0%). As for the future course format, small classes during residency training were the most popular and accounted for 29.1% of the participants. Compulsory theory courses (24.0%) ranked second in the list, followed by lectures (15.5%).

## Discussion

The results of this study show that knowledge of OSA among residents was relatively acceptable. Specifically, the accuracy percentage of knowledge on the OSA treatment still needs to be improved. Unexpectedly, despite positive attitudes, the majority of residents in this study reported a generalized lack of confidence in identifying and managing OSA. Further investigation finds that OSA training experience increases knowledge, but not confidence, and the current form of sleep medicine course is not ideal. This finding is worrisome since physicians don't have confidence, let alone patients, resulting in low treatment adherence. Different from previous studies, our results emphasize the core deficiencies in medical education and remind substantial public health risks.

In this study, we found that the mean knowledge score was 12.6 (±2.7), with a mean correct rate of 70%. To better assess our results, a comparison to previous studies is also shown as follows. In Nigeria, the mean knowledge score of resident doctors in Internal Medicine was 10.7 (59.4%) (24), and internists from the United States (Washington University Physicians Network) was 13.3 (72.2%) (13). The results of the present study lie midway between the two literature results. For primary care physicians, the mean rate of correct varied from Latin American (25) (60%) to the Middle East and North Africa regions (70%) (26). These study results reveal the highly heterogeneous geographical heterogeneity which may be explained due to differences in sleep medicine education and training. There are also differences in the knowledge scores in different medical specialties. The mean rate of correction is around 55% among speech-language pathologists and dentists (27, 28). Also, Years of medical training appeared to be associated with an increase in correct responses rate, from recent graduates (53.5%) to practicing physicians (60.4%) (29). Nigerian graduating medical students reported the lowest score (42 %) (30). Putting our results in a global context, knowledge of OSA among residents was relatively acceptable since the correct rate is close to the highest score (US 72.2%) and higher than the lowest score reported so far (13).

When compared with other domestic studies, the mean knowledge score of the residents in the resident training program is higher than that of general practitioners in community medical institutions but still lower than that of specialist physicians, such as otolaryngologists and pulmonary physicians (31, 32). Regretfully, none of the residents answered all the questions correctly. When it comes to each problem, the lowest correct answer in this study was regarding laser-assisted uvuloplasty as an appropriate treatment for severe OSA (5.9%). Only 29.4% of the residents correctly answered the question “uvulopalatopharyngoplasty is an effective therapy for most OSA patients.” Comparing the results of other studies, the correct rate for the same question is 64% in North Africa and 81.8% in the United States (13, 26). Less than half of the residents correctly answered the question “CPAP therapy may lead to nasal congestion” (32.0%). These results suggested that there was a lack of knowledge among residents regarding OSA treatment in the present study. Consistent with previous studies, most physicians ranked surgical treatment as more important than CPAP therapy (25). As the first-line therapy for OSA, CPAP adherence in China has been relatively much lower than that in Western countries (33). Insufficient and incorrect knowledge of OSA treatment may influence the patient's choice of therapy. Despite the highly educated majority (Master's degree or higher), nearly two-thirds of the residents had not been exposed to any courses about OSA. Indeed, we have observed significant differences between participants with and without OSA training in mean knowledge scores. Similar statistical differences were also observed in age and level of training, which further confirms the role of clinical experience in improving OSA knowledge.

The mean attitude score was 3.6 (±0.6). We found that over 90% of the residents considered both OSA as a clinical disorder and identifying possible OSA patients very/extremely important. None considered these two aspects unimportant. However, only a minority of the residents either strongly agreed or agreed that they had confidence in identifying patients at risk for OSA (38%). Even with higher knowledge scores, the confidence is generally lower than primary care physicians in Latin America (73.5%), internal medicine residents in Nigeria (72%), and even graduating medical students (41%) (24, 25, 30). When it comes to confidence in managing OSA patients or CPAP therapy, the favorable proportion (strongly agreed or agreed) was even lower than 30%. In contrast to previous studies conducted in other countries, our results showed lower confidence despite the leading knowledge and attitude score. Lack of confidence in identifying potential OSA patients can further aggravate the underdiagnosis and misdiagnosis, and these patients cannot be referred promptly, leading to delays in treatment. Also, the ability of OSA diagnosis and treatment is of equal importance. Although traditionally suspected OSA patients were referred to a sleep specialist, the current diagnosis and treatment journey is cumbersome, time-consuming, and often frustrating, clearly affecting treatment adherence (3). In contrast to the increasing trend of OSA prevalence, workforce shortages in sleep medicine are expected to become more severe in the coming years (34). Since it is assumed that the influx of new sleep physicians is far from sufficient to replace those who are retiring. As OSA is a common chronic condition in need of a comprehensive chronic condition management approach, the current disease management pattern is not enough and more doctors are needed to participate in the management of OSA (9). It is necessary to strengthen the training and education of residents and help them increase confidence in treating OSA patients. On the one hand, increasing resident exposure may affect subspecialty choice and help to improve the current workforce shortage in sleep medicine. On the other hand, residents' complete knowledge and confident attitudes can help provide adequate patient education and increase patients' confidence and long-term compliance (35).

Junior residents had a significantly lower average knowledge score than senior residents. Residents who attended OSA training or treated OSA patients in the past had higher knowledge scores. Only 38.6% of the residents denied previous exposure to any OSA patients. Likewise, their mean knowledge scores were significantly reduced compared with those who had experience in managing OSA patients. Although the clinical management of OSA patients seemed effective in improving OSA knowledge, our analysis showed that a certain number of physicians (26.8%) answered “unsure” on the identification of patients, indicating a not optimistic situation and the possibility of missed diagnosis. No significant differences were detected between the above factors and attitude scores except for age. Further, we explored the independent determinants of the knowledge and confidence scores. Unexpectedly, OSA training is an important factor positively correlated with knowledge scores but not confidence scores. This reflects that current education can improve knowledge, but not confidence in OSA among residents.

The ability of physicians regarding OSA is a critical factor that influences the clinical suspicion of OSA and the likelihood of making appropriate referrals (36). Lack of physician knowledge about OSA leads to a misdiagnosis, missed diagnosis, and a delay in treatment. Untreated OSA is associated with the development of certain comorbid conditions and mortality, increased incidence of vehicular accidents, and reduced longevity (14, 37). With the high prevalence of sleep-disordered breathing recorded, OSA has increasingly become a public health concern (38). In the present, most surveys on current sleep medicine education reported are not satisfactory. In particular, for residency training programs, there are no unified curriculums or requirements so far. Strengthening residency education in OSA would help reduce the disease burden and prevent long-term complications. From a clinical perspective, increasing resident exposure to residents with board certifications in sleep medicine or sleep fellowship training would significantly improve the breadth of resident experience in the evaluation and treatment of sleep-related disorders. From didactic perspective, as exposure is associated with subspecialty choice, increasing resident exposure may help to improve the current shortage of sleep specialists/clinics.

Given the current condition of low confidence and lack of relevant OSA training in resident physicians, we further explored the effective training forms to improve future medical education. In the additional component of this survey, the willingness to participate in sleep breathing disorders-related courses and their specific forms were investigated. Almost all residents enthusiastically agreed that sleep breathing disorders should be included in medical education. As to specific course forms, small classes during residency training were chosen as the first choice instead of compulsory theory courses. Small classes during clinical rotation can provide case-based learning and help to apply the theory to clinical practice. Besides, this course form may be more interactive and thereby provide a better understanding of CPAP treatment (39). Compared with traditional courses, small classes have the advantages of flexible time, short duration, and high participation, especially during clinical rotations. Since Chinese resident physicians have a heavy clinical workload, this kind of active learning is more effective and was most favored by Chinese resident physicians in the present study. Thus, in order not to increase the additional burden on resident physicians, we recommend the provision of a course format change in the future. Moreover, open access to residency programs to sleep centers where infrastructure exists to deliver a clinical experience in sleep disorders should be encouraged. On-site teaching regarding OSA knowledge can make it vivid and easier to comprehend. Additionally, more creative mechanisms to enhance OSA training are needed. For instance, remote education can be used as an effective strategy to provide sleep medicine courses. This allows residents to learn during the fragmented time.

There are strengths and limitations to the present study. This was the first study to evaluate the knowledge and attitudes of OSA among residents in residency training programs in China. Conducted in the top-ranked residential training hospitals, the results are representative, residents here lack confidence, let alone in the country. Another strength of this study was the exploration of specific forms of the sleep-breathing disorder course, providing important references for future residency training. The limitations of this study included its relatively smaller sample size. Additionally, the confidence of residents in the OSAKA sleep questionnaire may not necessarily match competence in clinical work. Thus, practice questions need to be evaluated in future studies.

## Conclusion

This study revealed that despite adequate OSA knowledge among residents, they still have low confidence in OSA management. OSA training can only positively affect knowledge scores but not confidence scores. This reflects that current education does not increase physicians' confidence in the management of patients with OSA, which may affect subsequent treatment compliance and pose an underappreciated public health risk. More attention should be paid to strengthening residency education on OSA management, helping reduce the disease burden, and preventing long-term complications. In the future, simply adding courses is far from enough, improvements in content and form focusing on enhancing the physicians' confidence are strongly warranted.

## Data availability statement

The original contributions presented in the study are included in the article/Supplementary material, further inquiries can be directed to the corresponding author/s.

## Ethics statement

The studies involving human participants were reviewed and approved by the Medical Ethics Committee of Peking Union Medical College Hospital (Approval No. S-K 954). The patients/participants provided their written informed consent to participate in this study.

## Author contributions

RC and JL conceived and designed the study. RC and LS acquired the data. LS analyzed the data and drafted the manuscript. YX and JL critically revised the manuscript. All authors contributed to the article and approved the submitted version.

## Funding

This study was supported by the National Natural Science Foundation of China (81570085), the National High Level Hospital Clinical Research Funding (2022-PUMCH-B-106), and the CAMS Innovation Fund for Medical Sciences (CIFMS) (2021-I2M-C&T-B-013).

## Conflict of interest

The authors declare that the research was conducted in the absence of any commercial or financial relationships that could be construed as a potential conflict of interest.

## Publisher's note

All claims expressed in this article are solely those of the authors and do not necessarily represent those of their affiliated organizations, or those of the publisher, the editors and the reviewers. Any product that may be evaluated in this article, or claim that may be made by its manufacturer, is not guaranteed or endorsed by the publisher.
