# Supplementary material for: Current medical education improves OSA-related knowledge but not confidence in residents: An underappreciated public health risk
Source: Front Psychiatry. 2022 Nov 11;13:973884. doi: 10.3389/fpsyt.2022.973884 (PMC9691645; doi:10.3389/fpsyt.2022.973884)
Supplement: Supplementary file 1 [file Table_1.DOCX]

Supplementary Material

**TABLE S1** | The characteristics of knowledge score and respondents' OSA training experience.

| **Knowledge** | **With OSA Training**  **Mean±SD** | **Without OSA Training**  **Mean±SD** | **Range** | ***p*-value** |
| --- | --- | --- | --- | --- |
| **Epidemiology** | 0.73±0.28 | 0.68±0.26 | 0-1 | 0.248 |
| **Pathophysiology** | 0.89±0.21 | 0.79±0.26 | 0-1 | **0.015** |
| **Symptoms** | 0.94±0.12 | 0.85±0.18 | 0-1 | **0.001** |
| **Diagnosis** | 0.79±0.21 | 0.75±0.23 | 0-1 | 0.214 |
| **Treatments** | 0.40±0.23 | 0.32±0.22 | 0-1 | **0.022** |

SD, standard deviation. Significant differences are in bold (two-sample t-test).
