# Supplementary material for: Current medical education improves OSA-related knowledge but not confidence in residents: An underappreciated public health risk
Source: Front Psychiatry. 2022 Nov 11;13:973884. doi: 10.3389/fpsyt.2022.973884 (PMC9691645; doi:10.3389/fpsyt.2022.973884)
Supplement: Supplementary file 2 [file Table_2.DOCX]

Supplementary Material

**TABLE S2 |** Specific items of attitudes questions and the proportion of answers.

| **OSA attitude** | **N (%)** |
| --- | --- |
| **As a clinical disorder, OSA is (Mean (SD))** | 4.3 (0.6) |
| Not important/somewhat important | 0 (0) |
| Important | 15 (9.8) |
| Very important/extremely important | 138 (90.2) |
| **Identifying patients with OSA is (Mean (SD))** | 4.4 (0.6) |
| Not important/somewhat important | 0 (0) |
| Important | 11 (7.2) |
| Very important/extremely important | 142 (92.8) |
| **Confidence in Identifying at-risk patient (Mean (SD))** | 3.3 (0.9) |
| Strongly disagree/disagree | 28 (18.3) |
| Neither agree nor disagree | 67 (43.8) |
| Agree/strongly agree | 58 (38.0) |
| **Confidence in Managing patients with OSA (Mean (SD))** | 3.1 (1.0) |
| Strongly disagree/disagree | 38 (24.8) |
| Neither agree nor disagree | 73 (47.7) |
| Agree/strongly agree | 42 (27.5) |
| **Confidence in Managing patients on CPAP (Mean (SD))** | 3.0 (1.1) |
| Strongly disagree/disagree | 44 (28.7) |
| Neither agree nor disagree | 69 (45.1) |
| Agree/strongly agree | 40 (26.2) |
| **Average** **attitude score (Mean (SD))** | 3.6(0.6) |
